# Protocol for assessing distances in pathway space for classifier feature sets from machine learning methods

**DOI:** 10.1016/j.xpro.2025.103681

**Published:** 2025-03-18

**Authors:** Bahar Tercan, Victor H. Apolonio, Vinicius S. Chagas, Christopher K. Wong, Jordan A. Lee, Christina Yau, Christopher C. Benz, Joshua M. Stuart, Brian J. Karlberg, Kyle Ellrott, Jasleen K. Grewal, Steven J.M. Jones, The Cancer Genome Atlas Analysis Network, Theo A. Knijnenberg, Mauro A.A. Castro, Vinicius S. Chagas, Victor H. Apolonio, Verena Friedl, Joshua M. Stuart, Vladislav Uzunangelov, Christopher K. Wong, Rameen Beroukhim, Andrew D. Cherniack, Galen F. Gao, Gad Getz, Stephanie H. Hoyt, Xavier Loinaz, Whijae Roh, Chip Stewart, Lindsay Westlake, Christopher C. Benz, Jasleen K. Grewal, Steven J.M. Jones, A. Gordon Robertson, Samantha J. Caesar-Johnson, John A. Demchok, Ina Felau, Anab Kemal, Roy Tarnuzzer, Peggy I. Wang, Zhining Wang, Liming Yang, Jean C. Zenklusen, Rehan Akbani, Bradley M. Broom, Zhenlin Ju, Andre Schultz, Akinyemi I. Ojesina, Katherine A. Hoadley, Avantika Lal, Daniele Ramazzotti, Chen Wang, Alexander J. Lazar, Lewis R. Roberts, Bahar Tercan, Taek-Kyun Kim, Ilya Shmulevich, Paulos Charonyktakis, Vincenzo Lagani, Ioannis Tsamardinos, Esther Drill, Ronglai Shen, Martin L. Ferguson, Kami E. Chiotti, Kyle Ellrott, Brian J. Karlberg, Jordan A. Lee, Eve Lowenstein, Paul T. Spellman, Adam Struck, Christina Yau, D. Neil Hayes, Toshinori Hinoue, Hui Shen, Peter W. Laird, Jean C. Zenklusen, A. Gordon Robertson, Peter W. Laird, Andrew D. Cherniack, Mauro A.A. Castro

**Affiliations:** 1 Institute of Systems Biology, 401 Terry Avenue North, Seattle, WA 98109, USA; 2 Bioinformatics and Systems Biology Laboratory, Federal University of Paraná, Curitiba, PR 81520-260, Brazil; 3 UC Santa Cruz Genomics Institute and Department of Biomolecular Engineering, Santa Cruz, CA 95060, USA; 4 Oregon Health and Science University, Portland, OR 97239, USA; 5 University of California, San Francisco, Department of Surgery, San Francisco, CA 94158, USA; 6 Buck Institute for Research on Aging, Novato, CA 94945, USA; 7 Canada’s Michael Smith Genome Sciences Centre, BC Cancer, Vancouver, BC, Canada; 8 Center for Cancer Genomics, National Cancer Institute, Bethesda, MD 20892, USA; 9 Department of Epigenetics, Van Andel Institute, Grand Rapids, MI 49503, USA; 10 The Broad Institute of Harvard and MIT, Cambridge, MA 02142, USA; 11 Department of Medical Oncology, Dana-Farber Cancer Institute, Boston, MA 02215, USA; 12 Harvard Medical School, Boston, MA 02115, USA

**Keywords:** bioinformatics, computer sciences, systems biology

## Abstract

As genes tend to be co-regulated as gene modules, feature selection in machine learning (ML) on gene expression data can be challenged by the complexity of gene regulation. Here, we present a protocol for reconciling differences in classifier features identified using different ML approaches. We describe steps for loading the PathwaySpace R package, preparing input for analysis, and creating density plots of gene sets. We then detail procedures for testing whether apparently distinct feature sets are related in pathway space.

For complete details on the use and execution of this protocol, please refer to Ellrott et al.^1^

## Highlights

• Protocol to test whether distinct gene sets reflect related biology
• Steps for building a pathway space for graph- or network-based distance analysis
• Instructions for calculating a pathway distance metric for any pair of gene sets
• Guidance on exploring relationships between gene sets in pathway space

## Abstract (editor highlights)

Publisher’s note: Undertaking any experimental protocol requires adherence to local institutional guidelines for laboratory safety and ethics.

## Graphical abstract

**Figure:**
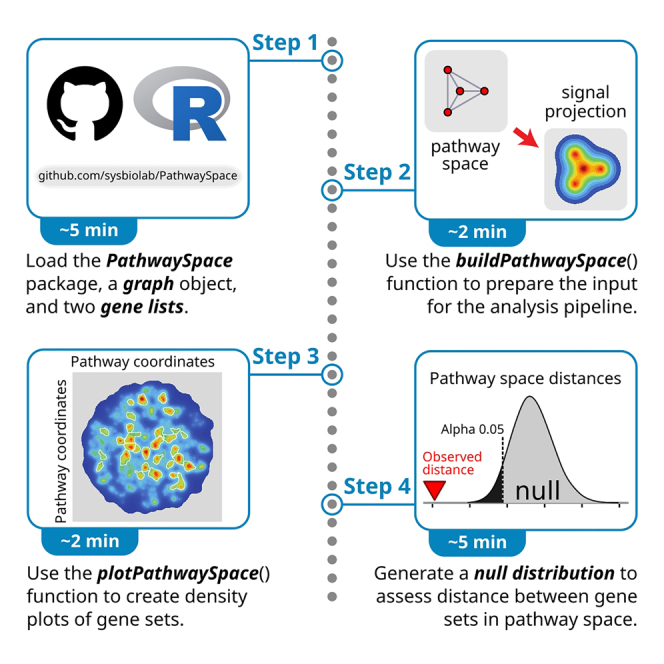

## Before you begin

### Overview

We generated a general method by which gene features that are identified through machine learning (ML) algorithms can classify non-TCGA tumors into TCGA subtypes.^1^ We found that ML algorithms often produced non-overlapping classifier feature sets that had similar accuracy profiles. We showed that distinct ML classifier features tended to reflect coregulated genes from the same pathways, and the pathways carry comparable biological information for subtype classification. Here, we present a protocol that allows comparing pathway distances between lists of genes. We describe steps involved in constructing a two-dimensional (2D) 'pathway space' in order to visualize and quantify the degree to which different gene sets are related. The *R* package *PathwaySpace* provides the data and source code required to execute all steps of this protocol.

### The pathway distance problem

**Figure 1:**
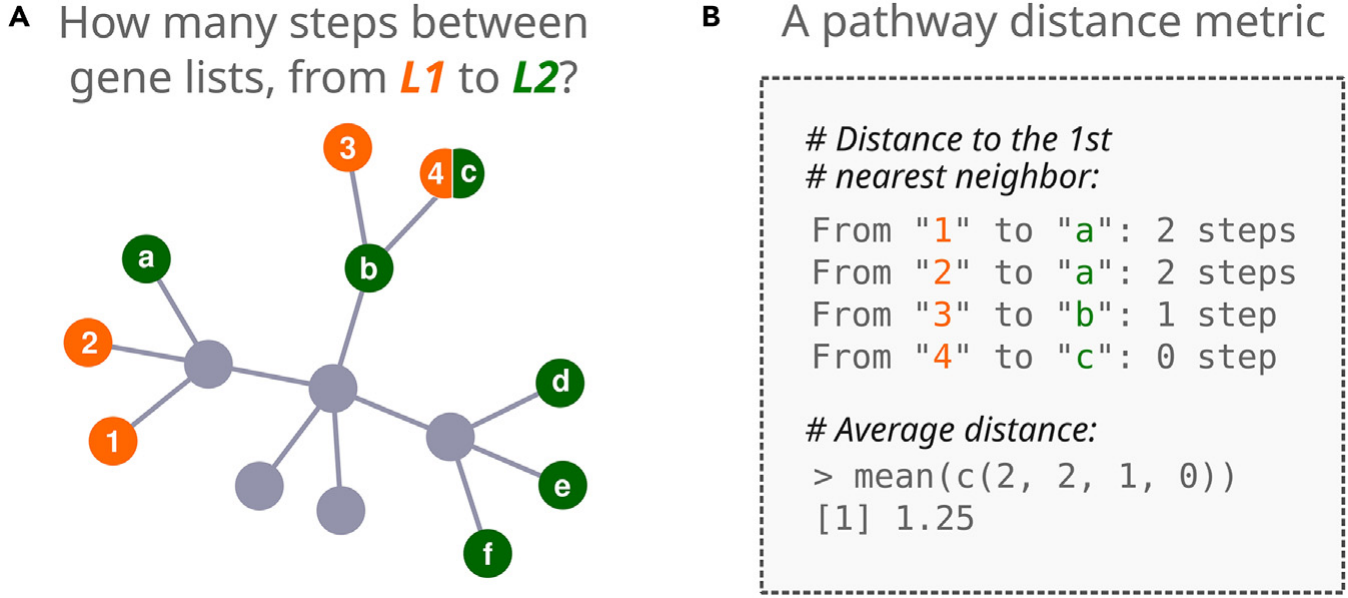
The pathway distance problem addressed by the *PathwaySpace* package (A) A schematic pathway space with 'toy' gene lists *L1* and *L2*. The package addresses the question: “How many steps are required to 'walk' from *L1* to *L2*?”. (B) The average path distance to the first nearest neighbors, between gene lists *L1* and *L2*, is 1.25.

For this protocol, we implemented a new metric to statistically assess distances between vertices. As an example, we introduce this metric in Figure 1A, presenting both a schematic pathway space graph and two hypothetical gene lists, *L1* and *L2.* We calculate a distance between these lists as the average path distance to first nearest neighbors (Figure 1B). In the protocol that follows, while there is minimal overlap between genes in *L1* and *L2*, we test whether these two gene lists are positioned more closely to each other in pathway space than we would expect by chance.

### Computational requirement

Hardware: RAM >= 16 GB.

Software: R (>=4.4), RStudio and PathwaySpace.

### Software installation

**Timing: ∼30 min**

In this step, we download and install *R* and *RStudio*, followed by the *PathwaySpace* package.

1. Download and install *R* and *RStudio*.
  a. To download R: https://cran.r-project.org/
  b. To download RStudio: https://rstudio.com/products/rstudio/
2. Download and install *PathwaySpace* in *RStudio*:

> install.packages("PathwaySpace")

**Note**: Additional *R* dependencies will be automatically downloaded during installation, including *RGraphSpace*, *igraph*, *ggplot2*, *ggrepel*, *RANN*, and *scales* packages.

## Key resources table

**Table:** 

REAGENT or RESOURCE	SOURCE	IDENTIFIER
**Deposited data**		

Pruned Pathway Commons v.12	Ellrott et al.^1^	https://gdc.cancer.gov/about-data/publications/CCG-TMP-2022
PathwaySpace source	This paper	https://github.com/sysbiolab/PathwaySpace

**Software and algorithms**		

R 4.4.1	CRAN	https://cran.r-project.org
RStudio v.2023.06.1	Posit	https://posit.co/products/open-source/rstudio/
PathwaySpace v.1.0.0	CRAN	https://cran.r-project.org/package=PathwaySpace
RGraphSpace v.1.0.7	CRAN	https://cran.r-project.org/package=RGraphSpace
igraph v.2.0.3	CRAN	https://cran.r-project.org/package=igraph
ggplot2 v.3.5.1	CRAN	https://cran.r-project.org/package=ggplot2
ggrepel v.0.9.5	CRAN	https://cran.r-project.org/package=ggrepel
RANN v.2.6.2	CRAN	https://cran.r-project.org/package=RANN
scales v.1.3.0	CRAN	https://cran.r-project.org/package=scales

## Step-by-step method details

### Loading packages and datasets

**Timing: ∼5 min**

In this step we load the required packages and datasets, and then display a pre-processed *igraph*^2^ object with a previously defined layout.^1^ Attributes of vertices include coordinates (i.e., *x* and *y*) and a 'name'. The 'name' attribute is provided as a gene symbol, and the graph is derived from the Pathway Commons (version 12).^3^ For a detailed description, please see Ellrott et al.^1^

1. Load the required packages.

> library(PathwaySpace)

> library(RGraphSpace)

> library(ggplot2)

> library(igraph)

2. Load the *igraph* object derived from Pathway Commons V12.

> data("PCv12_pruned_igraph", package = "PathwaySpace")

3. Check number of vertices.

> length(PCv12_pruned_igraph)

# [1] 12990

4. Check vertex names.

> head(V(PCv12_pruned_igraph)$name)

# [1] "A1BG" "AKT1" "CRISP3" "GRB2" "PIK3CA" "PIK3R1"

5. Get top-connected nodes for visualization.

> top10hubs <- igraph::degree(PCv12_pruned_igraph)

> top10hubs <- names(sort(top10hubs, decreasing = TRUE)[1:10])

> head(top10hubs)

# [1] "GNB1" "TRIM28" "RPS27A" "CTNNB1" "TP53" "ACTB"

**Figure 2:**
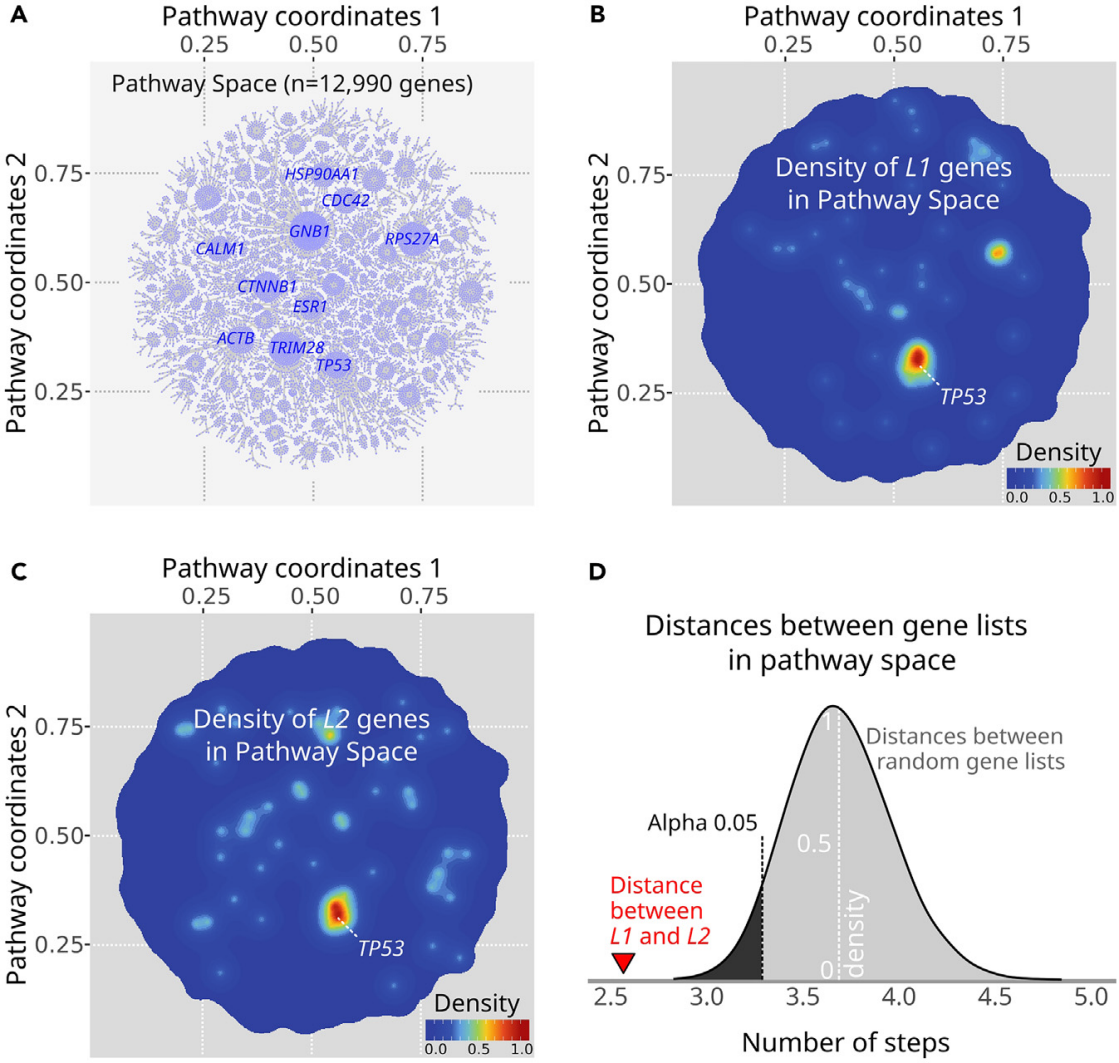
Assessing distances between gene lists in pathway space (A) Graph derived from the Pathway Commons V12.^1^^,^^3^ (B) Density of *L1* genes in pathway space. (C) Density of *L2* genes in pathway space. (D) Distances between gene lists, in terms of the number of steps required to 'walk' from *L1* to *L2* (red triangle). The bell-shaped curve represents the null distribution for pathway distances calculated between random gene lists that have the same lengths as *L1* and *L2*. The left region (in black) denotes the range of values where the null hypothesis is rejected for alpha <= 0.05.

***Note:*** The *plotGraphSpace*() function will display the layout of a graph derived from the Pathway Commons V12, as depicted in Ellrott et al.^1^ (Figure 2A). In the image, we annotate the top 10 'hub' genes (*i.e.* the genes with the highest degree centrality).

6. Display the graph layout labeled with the *top10hubs* (Figure 2A).

> plotGraphSpace(PCv12_pruned_igraph, marks = top10hubs, mark.color = "blue")

### Building a *PathwaySpace* object

**Timing: ∼2 min**

Next, we will run the *buildPathwaySpace*() constructor to generate a *PathwaySpace* object. To illustrate the *PathwaySpace* distance metric, we will also prepare two gene lists, each drawn from the same functional annotation. For this demonstration, we will use the MSigDB^4^ P53 Hallmark gene set as our reference for functional annotation.

7. Run the *PathwaySpace* constructor.

> pspace_PCv12 <- buildPathwaySpace(g = PCv12_pruned_igraph)

8. Load a list with Hallmark gene sets.

> data("Hallmarks_v2023_1_Hs_symbols", package = "PathwaySpace")

9. Intersect Hallmarks with the 'PathwaySpace' object.

> hallmarks <- lapply(hallmarks, intersect, y = names(pspace_PCv12) )

10. Get the 'HALLMARK_P53_PATHWAY' gene set for demonstration.

> HALLMARK_P53_PATHWAY <- hallmarks$HALLMARK_P53_PATHWAY

> length(HALLMARK_P53_PATHWAY)

# [1] 173

11. Extract two gene lists each with 50 genes.

> set.seed(12)

> L1 <- sample(HALLMARK_P53_PATHWAY, 50)

> set.seed(34)

> L2 <- sample(HALLMARK_P53_PATHWAY, 50)

12. Check gene list sizes and intersection.

> length(L1)

# [1] 50

> length(L2)

# [1] 50

> length(intersect(L1, L2))

# [1] 11

***Note:*** Since, here, we draw *L1* and *L2* from the same gene list, we expect that the lists will intersect to some degree, but the overlap, which we will assess with the *phyper*() function, may not be, and is not, statistically significant (*P* = 0.86, one-sided hypergeometric test).

13. Use the *phyper*() function to assess the overlap between *L1* and *L2.*

> phyper(q = 11, m = 50, n = 173-50, k = 50, lower.tail = FALSE)

# [1] 0.86 ## this is a p-value

***Note:*** Alternatively, for demonstration purposes we provide these gene lists as a flat file (see supplemental information). To try this, download the 'csv' file from 'DataS1'. Then, load this file into your *R* session.

14. For loading the gene lists *L1* and *L2* from a flat file.

> GeneList <- read.csv(file = "DataS1.csv", header = TRUE)

> L1 <- GeneList$ID[GeneList$List == "L1"]

> L2 <- GeneList$ID[GeneList$List == "L2"]

15. When loading user’s gene lists, ensure the pathway annotation is consistent with the input data. For example, use the *all*() function to check if all genes are listed in the *PathwaySpace* object.

> all(L1 %in% names(pspace_PCv12) )

# [1] TRUE

> all(L2 %in% names(pspace_PCv12) )

# [1] TRUE

### Visualizing gene lists in *PathwaySpace*

**Timing: ∼2 min**

We now pose the question: “Would L*1* and *L2* gene lists be statistically close to one another in pathway space?” To explore this, we will start by visualizing the density of *L1* and *L2* genes within the *PathwaySpace* object (Figures 2B and 2C).

16. Set '1' to *L1* genes and '0' for all the others; this will instruct the pipeline to evaluate the *L1* genes.

> vertexSignal(pspace_PCv12) <- 0

> vertexSignal(pspace_PCv12)[ L1 ] <- 1

17. Run the network signal projection.

> pspace_PCv12 <- circularProjection(pspace_PCv12)

> pspace_PCv12 <- silhouetteMapping(pspace_PCv12)

18. Display the density of *L1* genes in the *PathwaySpace* (Figure 2B).

> plotPathwaySpace(pspace_PCv12, marks = "TP53", mark.size = 2, theme = "th3", title = "Density of L1 genes in PathwaySpace")

19. Set '1' to *L2* genes and '0' for all the others; this will instruct the pipeline to evaluate the *L2* genes.

> vertexSignal(pspace_PCv12) <- 0

> vertexSignal(pspace_PCv12)[ L2 ] <- 1

20. Run the network signal projection.

> pspace_PCv12 <- circularProjection(pspace_PCv12)

> pspace_PCv12 <- silhouetteMapping(pspace_PCv12)

21. Display the density of *L2* genes in the *PathwaySpace* (Figure 2C).

> plotPathwaySpace(pspace_PCv12, marks = "TP53", mark.size = 2, theme = "th3", title = "Density of L2 genes in PathwaySpace")

### Assessing pathway space distances

**Timing: ∼5 min**

Finally, we will assess distances between gene lists in pathway space. The *pathDistances*() function will calculate the distance between the *L1* and *L2* gene lists, and between 10,000 random gene lists of the same size as *L1* and *L2*. The results are depicted in Figure 2D, which shows that the actual distance between *L1* and *L2* is significantly less than the *null* distances from the random gene lists.

22. Compute a vertex-wise distance matrix (i.e., distance between genes).

> gdist <- igraph::distances(graph = PCv12_pruned_igraph, algorithm = "unweighted")

23. Calculate distances between *L1* and *L2*, and between random gene lists.

> pdist <- pathDistances(gdist=gdist, from = L1, to = L2, nperm = 10000)

24. Plot observed and *null* distances (Figure 2D).

> plotPathDistances(pdist=pdist)

## Expected outcomes

The current protocol is expected to generate density plots for a given gene set in a pathway space, as shown in Figures 2B and 2C. This representation should be useful for visualizing density patterns on large graphs. A user should also be able to assess distances between two gene sets in pathway space, as shown in Figure 2D.

## Limitations

The *pathDistances*() function returns distances between two gene lists, within a pathway space. Pairwise comparisons could also be done between multiple gene lists, though this would require customizing the input data. To facilitate this, the *pathDistances*() function accepts a generic vertex-wise distance matrix. This flexibility is particularly useful, as it allows incorporating user-defined distance matrices calculated by other algorithms.

## Troubleshooting

### Problem 1

You may encounter problems in attempting to visualize very large *igraph* objects using standard *R* graphic devices.

### Potential solution

Use the *RGraphSpace* package to integrate *igraph* and *ggplot2* graphics. For more information, refer to the *RGraphSpace* documentation.

### Problem 2

You may encounter problems while automatically downloading and installing *R* dependencies.

### Potential solution

Use the key resources table to install each *R* package individually.

### Problem 3

You may not find the *PathwaySpace’*s vignette available on your *R* session.

### Potential solution

Go to the *PathwaySpace’*s GitHub repository for alternative installation options, at https://github.com/sysbiolab/PathwaySpace.

### Problem 4

You may need to compare pathway distances calculated from gene lists of different sizes.

### Potential solution

In this case, we recommend using distances that have been normalized as z-scores. The problem here is that the larger the sizes of any two gene lists, the fewer the number of steps required to walk from one to the other in pathway space. Therefore, distances between list pairs of different sizes must be normalized to make them comparable. A z-score normalization is available in the *pathDistances*() and *plotPathDistances*() functions.

### Problem 5

You may want to explore gene set distances using different graphs and null models.

### Potential solution

The rationale for the *PathwaySpace* distance null model was established in our primary research paper, Ellrott et al.,^1^ and this involved generating a random distribution of gene sets in a way that preserved the same basic properties of a gene set within a reference graph (e.g., the number of vertices, edges, and spatial constraints). As a reference graph for the current version of *PathwaySpace* we used the Pathway Commons (version 12).^3^ In order to use a different reference graph, we recommend assessing the stability of the null model under random perturbations. The supplemental information and Figure S1 provide a more in-depth discussion of this issue. In brief, these supplementary results indicate that the *PathwaySpace* distance null model is robust to missing information and capable of detecting short distances in sparse graphs that are enriched with true pathway-level associations.

## Resource availability

### Lead contact

Further information and reasonable requests for resources should be directed to and will be fulfilled by the lead contact, Mauro Castro (mauro.castro@ufpr.br).

### Technical contact

Technical questions on executing this protocol should be directed to and will be answered by the technical contact, Bahar Tercan (btercan@systemsbiology.org).

### Materials availability

No materials were generated in this study.

### Data and code availability

• All source code has been deposited in the *GitHub* repository and is publicly available at https://github.com/sysbiolab/PathwaySpace. The original version (v.1.0.0) of the *R* source code has been archived in the *CRAN* repository and is available at https://cran.r-project.org/package=PathwaySpace (https://doi.org/10.32614/CRAN.package.PathwaySpace).
• The PathwaySpace package is distributed under Artistic-2.0 License (https://www.r-project.org/Licenses/Artistic-2.0).
• Any additional information required to reanalyze the data reported in this paper is available from the lead contact upon reasonable request.

## Consortia

The members of the Cancer Genome Atlas Analysis Network are: Theo A. Knijnenberg, Mauro A. A. Castro, Vinicius S. Chagas, Victor H. Apolonio, Verena Friedl, Joshua M. Stuart, Vladislav Uzunangelov, Christopher K. Wong, Rameen Beroukhim, Andrew D. Cherniack, Galen F Gao, Gad Getz, Stephanie H. Hoyt, Xavier Loinaz, Whijae Roh, Chip Stewart, Lindsay Westlake, Christopher C. Benz, Jasleen K. Grewal, Steven J.M. Jones, A. Gordon Robertson, Samantha J. Caesar-Johnson, John A. Demchok, Ina Felau, Anab Kemal, Roy Tarnuzzer, Peggy I. Wang, Zhining Wang, Liming Yang, Jean C. Zenklusen, Rehan Akbani, Bradley M. Broom, Zhenlin Ju, Andre Schultz, Akinyemi I. Ojesina, Katherine A. Hoadley, Avantika Lal, Daniele Ramazzotti, Chen Wang, Alexander J. Lazar, Lewis R. Roberts, Bahar Tercan, Taek-Kyun Kim, Ilya Shmulevich, Paulos Charonyktakis, Vincenzo Lagani, Ioannis Tsamardinos, Esther Drill, Ronglai Shen, Martin L. Ferguson, Kami E. Chiotti, Kyle Ellrott, Brian J. Karlberg, Jordan A. Lee, Eve Lowenstein, Paul T. Spellman, Adam Struck, Christina Yau, D. Neil Hayes, Toshinori Hinoue, Hui Shen, Peter W. Laird.

## Acknowledgments

The authors would like to acknowledge the support of the National Cancer Institute. This work was funded through NIH/NCI grants (U24CA264029 to A.D.C., U24CA264023 to P.W.L., U24CA264007 to K.E., U24CA264009 to J.M.S., and 5U24CA210952-05 to S.J.M.J.) and Brazilian funding from CNPq (316622/2021-4; 440412/2022-6), CAPES (88882.632783/2021-01), and Fundação Araucária (NAPI Bioinformática) to M.A.A.C.

## Author contributions

Conceptualization: C.K.W., J.M.S., A.D.C., P.W.L., and M.A.A.C.; data curation: B.T., C.C.B., C.Y., V.H.A., K.E., J.K.G., B.J.K., and M.A.A.C.; formal analysis: V.H.A. and M.A.A.C.; funding acquisition: M.A.A.C., J.C.Z., A.D.C., and P.W.L.; investigation: B.T., C.K.W., V.H.A., V.S.C., and M.A.A.C.; methodology: B.T., A.G.R., V.H.A., V.S.C., and M.A.A.C.; project administration: M.A.A.C., A.G.R., J.C.Z., A.D.C., and P.W.L.; software: M.A.A.C., V.H.A., and V.S.C.; supervision: K.E., J.C.Z., P.W.L., J.M.S., A.D.C., and M.A.A.C.; validation: C.K.W., M.A.A.C., J.A.L., and B.T.; visualization: M.A.A.C., J.K.G., A.G.R., V.H.A., and B.T.; writing – original draft: M.A.A.C., A.G.R., and B.T.; writing – review and editing: B.T., P.W.L., A.G.R., A.D.C., and M.A.A.C.

## Declaration of interests

A.D.C. receives research support from Bayer and consults for KaryoVerse.

## Declaration of generative AI and AI-assisted technologies in the writing process

During the preparation of this work, the authors used ChatGPT (version 4, OpenAI) to improve readability of the R package’s documentation while using the RStudio Desktop (https://posit.co/). After using this tool/service, the authors carefully reviewed and edited the content as needed and take full responsibility for the content of the publication.

## Supplemental information

Document S1. Supplemental text and Figure S1, related to Problem 5

Data S1. Supplemental flat file with gene lists, related to Step 14
