# Supplementary material for: Protocol for assessing distances in pathway space for classifier feature sets from machine learning methods
Source: STAR Protoc. 2025 Mar 18;6(2):103681. doi: 10.1016/j.xpro.2025.103681 (PMC11968257; doi:10.1016/j.xpro.2025.103681)
Supplement: Document S1. Supplemental text and Figure S1, related to Problem 5 [file mmc1.pdf]

This PDF file includes:

- Supplemental Text

- Supplemental References

- Figure S1

Supplemental Text

The *PathwaySpace* distance null model

We implemented a distance null model to assess the statistical significance of distances between gene sets in a given graph. The rationale for the algorithm design was established in our primary research paper, Ellrott *et al.*,[1] and this involved generating a random distribution of gene sets in a way that preserved the same basic properties of a gene set within the reference graph (*e.g.* the number of vertices, edges, and spatial constraints), but without any underlying relationship that affected the distances between random gene sets. In this model, we computed distances between randomly distributed nodes, and compared observed distances in the actual graph to this random distribution. We then assessed statistical significance by evaluating whether the observed distances deviated significantly from the null distribution, using a p-value to determine whether the observed spatial relationships were unlikely to have arisen by chance. This approach provided a baseline to interpret the distances calculated in pathway space. In the following, we will provide additional details on model assumptions and strategies for evaluating the stability of the null model.

Model assumptions

The *PathwaySpace* distance null model assumes a specific metric, the average path distance to the first nearest neighbors, to measure distances between sets of features within a reference (pathway) graph. The null model assumes that the distances between such sets of nodes exhibit no inherent dependencies. This means that the placement of one node set does not influence the placement of another node set in the reference graph. The null model assumes that the reference graph size is sufficiently large to ensure that any random set of distances will accurately represent the broad range of possible outcomes. The model assumes that there is a shortest path between any pair of nodes in the reference graph, *i.e.* that there is only a single graph. Sub-graphs are not modeled in the distance null model, which is pruned for retaining a single main connected component; see Ellrott *et al.*[1] for additional details on the reference graph construction (sections *generating* and *visualizing* pathways).

Assessing stability of the null model

The stability of the distance null model depends on several factors. These include the model assumptions, any spatial constraints, the graph size, the choice of distance metric, and the method used to generate the random distribution. For *PathwaySpace*, the distance null model takes into account the observed graph structure of the Pathway Commons (version 12),[3] a large collection of biological pathways that we use as a reference graph. The graph contains genes (nodes) and associations between genes (edges). We assessed the stability of the null model by applying random perturbations to the reference graph, aiming to disrupt the underlying spatial constraints. We generated five types of random perturbations: permuting nodes, disconnecting nodes, disconnecting hubs, removing edges, and rewiring edges (**Figure S1**). We apply each of these perturbations at increasing intensities, ranging from minor to major random changes, until the perturbed graph no longer preserves similarity with the reference (unperturbed) graph. We created null distributions by assessing pathway space distances between random gene lists, and we assessed pathway space distances between two actual gene lists, *L1* and *L2*, which we constructed as mutually exclusive sets of neighboring nodes in the reference graph. These two gene lists are one step apart from each other in the reference graph, representing a true short distance; we expect that *L1* and *L2* will move further apart as the reference graph changes due to the random perturbations. We designed this analysis pipeline to address two questions: **1**) Will the distance metric remain stable under the different perturbations? **2**) Will the *PathwaySpace* distance null model discriminate the *L1*-to-*L2* distance from the random null distances?

In **Figure S1A** we show that the null distances are highly stable to perturbations applied to nodes, and that the null model is able to discriminate the *L1*-to-*L2* distance in the perturbed graphs that preserve at least 60% similarity with the reference graph (red-filled triangles). However, this type of perturbation does not alter the graph structure; it merely permutes nodes, while maintaining the same wiring configuration, thereby preserving the relative distances between random gene sets. The loss of spatial relation between *L1* and *L2* in highly-perturbed graphs demonstrates that the randomization method is efficient in disrupting dependencies between sets of nodes (genes).

In **Figure S1B** we show perturbation types that affect the graph's wiring configuration, by either rewiring (left panel) or by removing edges (right panel). These results show that the null distances are affected by perturbations that lead to changes in wiring configurations, which can be explained by the disruption of paths that once connected nearby nodes, resulting in an overall increase in distances between gene sets. The *L1*-to-*L2* distance is discriminated from null distances in rewiring-perturbed graphs that preserve similarity at >=80%, and in all instances of the edge-removal perturbations (red-filled triangles). If we assume that the reference graph is enriched with true pathway-level associations,[3] rewiring edges of true associations should increase both false associations

and false non-associations, while removing edges should increase false non-associations only. Taken together, these results indicate that the *PathwaySpace* distance null model is robust to missing information and capable of detecting short distances in sparse graphs that are enriched with true pathway-level associations.

In **Figure S1C**, we assessed two additional types of graph perturbations by combining events that target both nodes and edges. Random perturbations that disconnect nodes (left panel) produced results similar to those obtained by removing edges, suggesting that the null model is also robust to missing nodes. However, this observation should be interpreted with caution, as biological pathways can exhibit complex configurations, including the presence of ‘hub’ genes (*i.e.* nodes that have a higher number of connections than most other nodes). Random perturbations targeting hub genes (right panel) caused instability in the pathway space distance metric, either having no effect or severely disrupting the calculated distances between the *L1* and *L2* gene lists. These results are consistent with observations that biological networks are robust to random errors, but vulnerable to preferential removal of important nodes, which may lead to network fragmentation [S1, S2]. For reproducing the results presented in **Figure S1**, refer to the *data and code availability* section.

Supplemental References

S1. Tanaka G, Morino K, Aihara K. Dynamical robustness in complex networks: the crucial role of low-degree nodes. Sci Rep. 2012;2:232. doi: 10.1038/srep00232. PMID: 22355746

S2. Albert R, Jeong H, Barabasi AL. Error and attack tolerance of complex networks. Nature. 2000;406(6794):378-82. doi: 10.1038/35019019. PMID: 10935628.

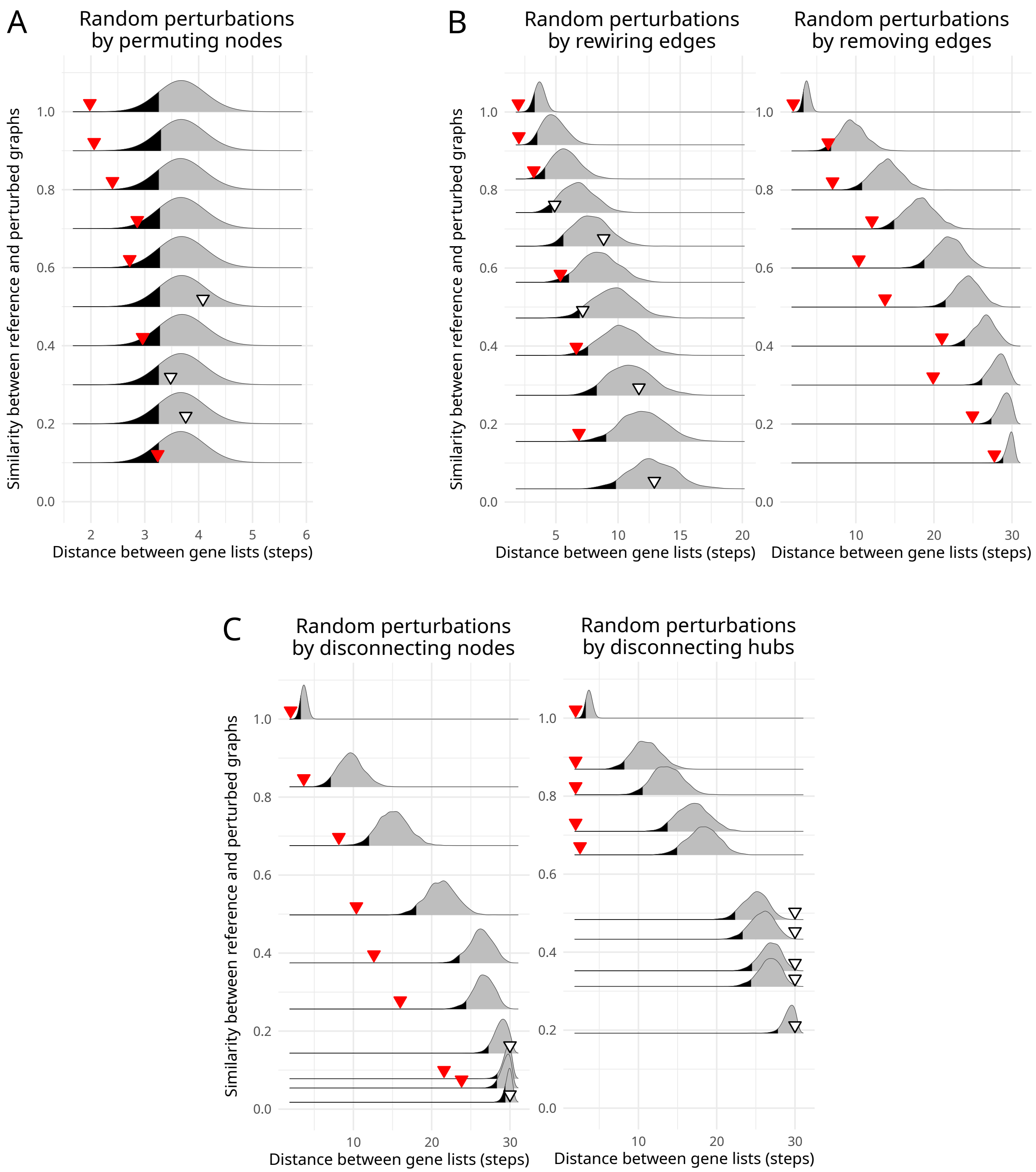

Figure S1. Assessing the *PathwaySpace* null distance model under different graph perturbations.
A) Random perturbation types applied to graph nodes. **B**) Random perturbation types applied to graph edges. **C**) Random perturbation types applied to graph nodes and edges. The bell-shaped curves represent null distributions under varying perturbation levels, indicated in the y-axis by the similarity between the perturbed and the reference (unperturbed) graphs. A similarity of '0.6' indicates that the perturbed graph preserves 60% of its identity with the reference graph. The left regions (black) indicate the range of values where the null hypothesis is rejected at $\alpha \leq 0.05$. Distances along the x-axis represent the number of steps between gene lists in pathway space. Distances between random gene lists are represented by the null distribution, while distances between actual gene lists (*i.e. L1* and *L2*; see protocol for details) are represented by triangles (red triangles are within the rejection region). The *L1* and *L2* gene lists are constructed as mutually exclusive sets of neighboring nodes in the reference graph. All perturbation analyses were conducted using the same *PathwaySpace* reference graph outlined in the *STAR Protocol* main document.
